# Association of H3K9me3 with breast cancer prognosis by estrogen receptor status

**DOI:** 10.1186/s13148-022-01363-y

**Published:** 2022-10-27

**Authors:** Meng Zhou, Jin-qi Yan, Qian-xin Chen, Yuan-zhong Yang, Yue-lin Li, Yue-xiang Ren, Zi-jin Weng, Xiao-fang Zhang, Jie-xia Guan, Lu-ying Tang, Ze-fang Ren

**Affiliations:** 1 School of Public Health, Sun Yat-sen University, 74 Zhongshan 2nd Rd, Guangzhou, 510080 China; 2 The Sun Yat-sen University Cancer Center, Guangzhou, China; 3 The First Affiliated Hospital, Sun Yat-sen University, Guangzhou, China; 4 The Third Affiliated Hospital, Sun Yat-sen University, 600 Tianhe Rd, Guangzhou, 510630 China

**Keywords:** Breast cancer, Prognosis, H3K9me3, Estrogen receptor

## Abstract

**Background:**

Cellular experiments revealed that a decreased histone H3 lysine 9 trimethylation (H3K9me3) level was associated with the upregulation of oncogenes in breast cancer cells. Moreover, the role of H3K9me3 in breast cancer was closely associated with estrogen receptor (ER) status. Therefore, we aimed to examine the prognostic value of H3K9me3 on breast cancer by ER status. The level of H3K9me3 in tumors were evaluated with tissue microarrays by immunohistochemistry for 917 women diagnosed with primary invasive breast cancer. Hazard ratios (HRs) and their 95% confidence intervals (CIs) for overall survival (OS) and progression-free survival (PFS) were estimated using Cox regression models. Interaction between H3K9me3 and ER on the prognosis was assessed on multiplicative scale.

**Results:**

The level of H3K9me3 in tumor tissues was lower than that in adjacent tissues. The high level of H3K9me3 was associated with a better OS (HR = 0.43, 95% CI: 0.21–0.86) and PFS (HR = 0.49, 95% CI: 0.29–0.81) among only ER-positive but not ER-negative tumors. Moreover, the interaction between the level of H3K9me3 and ER status (negative and positive) on the prognosis was significant (*P*_interaction_ = 0.011 for OS; *P*_interaction_ = 0.022 for PFS). Furthermore, the ER-positive tumors were stratified by ER-low and ER-high positive tumors, and the prognostic role of H3K9me3 was significant among only ER-high positive patients (HR = 0.34, 95% CI: 0.13–0.85 for OS; HR = 0.47, 95% CI: 0.26–0.86 for PFS).

**Conclusions:**

Our study showed that the prognostic value of H3K9me3 on breast cancer was related to ER status and expression level, and the high level of H3K9me3 was associated with a better prognosis among ER-positive tumors, particularly ER-high positive tumors.

**Supplementary Information:**

The online version contains supplementary material available at 10.1186/s13148-022-01363-y.

## Background

Breast cancer has been the leading cause of cancer death in females for the past decade [1–3]. In 2020, female breast cancer has surpassed lung cancer as the leading cause of global cancer incidence, representing 11.7% of all cancer cases, which further increased the treatment burden [3]. The current cure rates of breast cancer are highly dependent on the molecular subtype of the tumor and the stage at diagnosis, which, in some cases, do not result in satisfactory clinical outcomes [4]. Therefore, the search for new biomarkers with therapeutic purpose is still needed to assist in the clinical management of breast cancer.

Epigenetic changes such as DNA methylation, histone modification and microRNAs (miRNAs) play a crucial role in tumorigenesis and cancer progression [5–7]. It has been found that the level of many histone modification markers was associated with the prognosis of human cancers [8–11]. Among these markers, histone H3 lysine 9 trimethylation (H3K9me3) and histone H3 lysine 27 trimethylation (H3K27me3) were the hallmarks of repressive marker [12], and H3K9me3 was a key histone modification marker associated with the prognosis of many cancers [13–16], while one study found no association between the level of H3K9me3 and breast cancer survival [17]. However, cellular experiments revealed that H3K9me3 decreased during breast cancer transformation [18]. Moreover, a recent study found that decreased H3K9me3 level was associated with the upregulation of many oncogenes which were related to breast cancer prognosis [19].

We further noticed that the level of H3K9me3 was closely associated with estrogen receptor (ER) status (negative and positive) in breast cancer and the role of H3K9me3 depended on ER [20, 21], suggesting that the association of H3K9me3 with prognosis may be related to ER status. In addition, a recent update in the ASCO/CAP guideline recommends defining ER expression levels: negative, low level (1–10%) and high level (> 10%) depending on the proportion of positive tumor nuclei [22]. It has been found that ER-low positive patients revealed more similar clinicopathologic profiles to ER-negative rather than ER-high positive patients [23, 24]. It would be interesting to further examine the role of different expression levels of ER (low level and high level) on the association between H3K9me3 and the prognosis.

In the present study, therefore, we aimed to examine the prognostic value of H3K9me3 on breast cancer by ER status (negative vs. positive) and the different ER expression levels (ER-low positive vs. ER-high positive).

## Results

### Low level of H3K9me3 in breast cancer tissues

Of 917 women included in the analysis, almost all (99.0%) of them were pathologically diagnosed with invasive ductal carcinoma (IDC). The level of H3K9me3 in adjacent tissues was available in 633 patients, and the median (*P*_25_, *P*_75_) in tumor tissues [160.0 (62.5, 255.0)] was significantly lower than that in adjacent tissues [255.0 (210.0, 270.0)] (*p* < 0.001) (Table 1). When stratified by ER status, the level of H3K9me3 in tumor tissues was still lower than that in adjacent tissues for both ER-negative [210.0 (117.5.0, 255.0) vs 255.0 (180.0, 270.0), *p* < 0.001] and ER-positive [150.0 (56.7., 240.0) vs 255.0 (210.0, 270.0), *p* < 0.001] tumors.

**Table 1:** Level of H3K9me3 in tumor and adjacent tissues by ER status

H3K9me3	Total	*H*-score [median (*P*_25_, *P*_75_)]		*p* value^a^
		Tumor tissues	Adjacent tissues	
All	633	160.0 (62.5, 255.0)	255.0 (210.0, 270.0)	< 0.001
ER-negative	170	210.0 (117.5, 255.0)	255.0 (180.0, 270.0)	< 0.001
ER-positive	463	150.0 (56.7, 240.0)	255.0 (210.0, 270.0)	< 0.001

^a^*p* value for Wilcoxon signed rank test

### Demographic and clinicopathological characteristics and the associations with H3K9me3 in tumor tissues

The median age at diagnosis was 48 years (interquartile range: 41–56) among 917 eligible women and more than half (57.6%) of the women were premenopausal. The majority of the women were diagnosed with low histological grade (grade I/II: 73.3%), early clinical stage (stage I/II: 71.5%), ER-positive (73.1%), PR-positive (72.1%), or HER2-negative (66.8%) (Table 2). Univariate analysis showed that tumor size, nodal status, clinical stage and ER status were associated with OS and PFS (Additional file 1: Table S1).

**Table 2:** Demographic and clinicopathological characteristics and the associations with H3K9me3 [*N* (%)]

Characteristics	*N* (%)	*H*-score ≤ 240 (*n* = 667)	*H*-score > 240 (*n* = 250)	*p* value^a^
Age (years)				0.124
< 40	181 (19.7)	135 (20.2)	46 (18.4)	
40–60	606 (66.1)	447 (67.0)	159 (63.6)	
≥ 60	130 (14.2)	85 (12.7)	45 (18.0)	
Menopause				0.059
Pre-	506 (57.6)	380 (59.4)	126 (52.5)	
Post-	372 (42.4)	258 (40.6)	114 (47.5)	
Missing	39	29	10	
Histological grade				**0.020**
I/II	619 (73.3)	465 (75.5)	154 (67.5)	
III	225 (26.7)	151 (24.5)	74 (32.5)	
Missing	73	51	22	
Tumor size (cm)				0.605
≤ 2	278 (30.3)	199 (29.8)	79 (31.6)	
> 2	639 (69.7)	468 (70.2)	171 (68.4)	
Nodal status				**0.013**
Negative	412 (44.9)	283 (42.4)	129 (51.6)	
Positive	505 (55.1)	384 (57.6)	121 (48.4)	
Clinical stage				0.478
I	165 (18.0)	114 (17.1)	51 (20.4)	
II	491 (53.5)	359 (53.8)	132 (52.8)	
III	261 (28.5)	194 (29.1)	67 (26.8)	
ER				**0.001**
Negative	247 (26.9)	159 (23.8)	88 (35.2)	
Positive	670 (73.1)	508 (76.2)	162 (64.8)	
PR				** < 0.001**
Negative	256 (27.9)	165 (24.8)	91 (36.4)	
Positive	660 (72.1)	501 (75.2)	159 (63.6)	
Missing	1	1		
HER2				**0.006**
Negative	613 (66.8)	466 (69.9)	147 (58.8)	
Equivocal	76 (8.3)	52 (7.8)	24 (9.6)	
Positive	228 (24.9)	149 (22.3)	79 (31.6)	

^a^*p* value for chi-square test

The optimal cut-off value of H3K9me3 *H*-score was 240.0 according to the X-tile plot (Additional file 1: Fig. S2), and 667 (72.7%) patients had the *H*-score ≤ 240.0. Patients with the *H*-score ≤ 240.0 were more likely to have grade I/II, nodal positive, ER-positive, PR-positive and HER2-negative tumors than the subjects with *H*-score > 240.0 (Table 2). No marked differences in the level of H3K9me3 were observed between different age, menopausal status, tumor size and clinical stage.

### Association of H3K9me3 with breast cancer prognosis

Of the 917 eligible women, 127 died and 203 experienced disease progression with a median follow-up time of 85.2 months (interquartile range: 59.0–121.8). Five-year OS rate and PFS rate were 92.0% and 84.9%, respectively. In univariate analysis, no significant association was found between the level of H3K9me3 and breast cancer OS, while significant association (HR = 0.67, 95% CI 0.47–0.95) between a better breast cancer PFS and the high level of H3K9me3 was observed (Table 3). In multivariate analysis, a similar pattern of association was observed (HR = 0.70, 95% CI 0.49–0.99 for PFS).

**Table 3:** Univariate and multivariate association of H3K9me3 with breast cancer prognosis

H3K9me3	Total (%)	Events	Crude HR (95%CI)	*p* value	Adjusted HR (95%CI)^a^	*p* value^a^
OS				0.210		0.244
≤ 240.0	667 (72.7)	102	1.00 (reference)		1.00 (reference)	
> 240.0	250 (27.3)	25	0.76 (0.49, 1.17)		0.77 (0.49, 1.20)	
PFS				0.025		0.046
≤ 240.0	667 (72.7)	165	1.00 (reference)		1.00 (reference)	
> 240.0	250 (27.3)	38	**0.67 (0.47, 0.95)**		**0.70 (0.49, 0.99)**	

Significant results (*p* < 0.05) are shown in bold

^a^Adjusted for age at diagnosis, clinical stage, ER status, HER2 status

### Statistical interaction between ER status and H3K9me3

The results of stratified analysis by ER status showed that the prognostic value of H3K9me3 on breast cancer was significant only among patients with ER-positive tumors (Table 4). The high level of H3K9me3 was associated with a better breast cancer OS (HR = 0.43, 95% CI 0.21–0.86) and PFS (HR = 0.49, 95% CI 0.29–0.81) in ER-positive patients. Moreover, the interaction between the level of H3K9me3 and ER status on the prognosis was significant (*P*_interaction_ = 0.011 for OS; *P*_interaction_ = 0.022 for PFS).

**Table 4:** Associations of H3K9me3 with ER-negative and ER-positive breast cancer prognosis

H3K9me3	ER-negative		ER-positive		*P*_interaction_
	Events/total	Adjusted HR (95%CI)^a^	Events/total	Adjusted HR (95%CI)^a^	
OS					**0.011**
≤ 240.0	27/159	1.00 (reference)	75/508	1.00 (reference)	
> 240.0	16/88	1.41 (0.75, 2.65)	9/162	**0.43 (0.21, 0.86)**	
PFS					**0.022**
≤ 240.0	42/159	1.00 (reference)	123/508	1.00 (reference)	
> 240.0	21/88	1.10 (0.63, 1.84)	17/162	**0.49 (0.29, 0.81)**	

^a^Adjusted for age at diagnosis, clinical stage, HER2 status

Significant results (*p* < 0.05) are shown in bold

For patients with ER-positive tumors, stratified analysis by ER-low and ER-high positive tumors was conducted. Notably, the prognostic value of H3K9me3 was significant among patients with ER-high positive tumors (HR = 0.34, 95% CI 0.13–0.85 for OS; HR = 0.47, 95% CI 0.26–0.86 for PFS) (Table 5). However, no significant association was found among patients with ER-low positive tumors (HR = 0.64, 95% CI 0.21–1.91 for OS; HR = 0.53, 95% CI 0.20–1.38 for PFS).

**Table 5:** Associations of H3K9me3 with ER-low and ER-high positive breast cancer prognosis

H3K9me3	ER-low positive			ER-high positive		
	Events/total	Adjusted HR (95%CI)^a^	*P* value^a^	Events/total	Adjusted HR (95%CI)^a^	*P* value^a^
OS			0.419			**0.022**
≤ 240.0	21/114	1.00 (reference)		54/394	1.00 (reference)	
> 240.0	4/39	0.64 (0.21, 1.91)		5/123	**0.34 (0.13, 0.85)**	
PFS			0.192			**0.015**
≤ 240.0	31/114	1.00 (reference)		92/394	1.00 (reference)	
> 240.0	5/39	0.53 (0.20, 1.38)		12/123	**0.47 (0.26, 0.86)**	

Significant results (*p* < 0.05) are shown in bold

^a^Adjusted for age at diagnosis, clinical stage, HER2 status

## Discussion

In this study, we found that the level of H3K9me3 in breast cancer tissues was significantly lower than that in adjacent tissues. Moreover, the low level of H3K9me3 was associated with a poor breast cancer OS and PFS in ER-positive patients, while no significant association was found in ER-negative patients, and the interaction between H3K9me3 and ER on the prognosis was significant.

In consistent with our study, previous studies also found that the level of H3K9me3 was low in breast cancer tissues [18, 25]. The level of H3K9me3 in cells depended on histone lysine methyltransferases or the opposing demethylases, and various studies have shown that the demethylases KDM4A/JMJD2A, KDM4B/JMJD2B and/or KDM4C/JMJD2C are overexpressed in breast cancer [26]. In the present study, the decreased H3K9me3 level was observed for both ER-negative and ER-positive tumors when compared with the adjacent tissues, while the level of H3K9me3 in ER-positive tumors was lower than that in ER-negative tumors. It has been found that the histone demethylase KDM4B/JMJD2B is regulated by ERα [27, 28], which may explain that H3K9me3 level was lower in ER-positive tumors. In addition, we also showed that the low level of H3K9me3 was associated with low histological grade, nodal positive, PR-positive and HER2 negative tumors, while underlying mechanisms of these associations needs to be further explored.

Many cellular experiments revealed that a decreased level of H3K9me3 was associated with the overexpression of oncogenes; interestingly, some of these oncogenes were regulated by ER [19, 25, 27, 29]. These findings supported our result that the low level of H3K9me3 was associated with a poor prognosis and this association was significant only in ER-positive (particularly ER-high positive) patients but not in ER-negative patients. In addition, a previous population study found no significant association between H3K9me3 and breast cancer prognosis, which may be attributed to the high proportion (41.4%) of ER-negative patients [17].

Currently, epigenetic therapies are promising agents for overcoming clinical resistance to conventional treatments in breast cancer, but still not widely used [30, 31], and the further studies were needed. Our findings showed that the higher level of H3K9me3 was associated with a better prognosis of ER-positive (particularly ER-high positive) breast cancer. Combined with the related reports, inhibitors of H3K9me3 demethylase could be used for the treatment of ER-positive breast cancer [32, 33]. Interestingly, the H3K9me3 demethylase KDM4B/JMJD2B is regulated by ERα [27, 28], suggesting that the KDM4B/JMJD2B inhibitor would be more effective to improve the survival of ER-positive breast cancer patients.

Our study has several limitations that need to be taken into consideration. First, the IHC staining of H3K9me3 was evaluated by only one pathologist, which may lead to misclassification bias. However, the pathologist’s evaluation criteria are consistent; the relative relationship between all samples is almost unaffected. Therefore, even if the IHC staining was misclassified, the misclassification bias is likely to be non-differential and underestimate the true association. Second, only patients with tumor > 1 cm were included, which may lead to selective bias. However, the prognosis of patients with tumor ≤ 1 cm was excellent, even with less treatment [34]; thus, it is acceptable to select the patients with tumor > 1 cm as the study population. Third, the sample size of ER-low positive patients was small; the future study with larger sample size was more valuable. Finally, we didn not collect the information of treatment which was associated with the outcomes. However, since the treatment was determined according to the clinicopathological characteristics, and adjustment of these characteristics in the analysis was able to largely control the confounding effects of the treatment.

## Conclusions

In conclusion, this study firstly demonstrated that there was an interaction between H3K9me3 and ER on breast cancer prognosis and the prognostic value of H3K9me3 was significant among only ER-positive tumors, particularly ER-high positive tumors, but not ER-negative and ER-low positive tumors. Our study highlights H3K9me3 as a prognostic marker in ER-positive breast cancer and more investigations are expected to prove that H3K9me3 is a therapeutic target for ER-positive breast cancer.

## Methods

### Study population

A total of 1062 female patients with pathologically diagnosed primary invasive breast cancer and > 1 cm of tumor size in diameter between January 2008 and December 2015 were recruited from the Cancer Center of Sun Yat-sen University in Guangzhou, China. Patients with metastatic tumor and missing information of age at diagnosis, ER status and the level of H3K9me3 in tumor tissues (*N* = 129) were excluded (Additional file 1: Fig. S1). Of 933 eligible women, 917 (98.3%) were successfully followed up until Dec 31, 2021 and then were included in the statistical analysis. This study was approved by the Ethics Committee of the School of Public Health at Sun Yat-sen University. Informed consent was obtained from each participant.

### Baseline data collection

Information on demographic and clinicopathologic characteristics was collected at diagnosis from patients’ medical records, including age, menopausal status, clinical stage, histological grade, ER, progesterone receptor (PR) and human epidermal growth factor receptor 2 (HER2) status, etc. The definition of ER, PR and HER2 status was described in detail previously, and ER-positive was defined as ≥ 1% of tumor cell nuclei that are immunoreactive [35]. In the present study, furthermore, ER-low positive was defined as 1–10% of tumor cell nuclei that are immunoreactive; ER-high positive was defined as > 10% of tumor cell nuclei that are immunoreactive.

### Tissue microarray and immunohistochemistry

The level of H3K9me3 was evaluated with tissue microarrays (TMAs) by immunohistochemistry (IHC). TMAs were constructed as previously described [36]. The TMAs were baked at 60 °C for 2 h and then dewaxed with xylene and ethanol. Then antigen retrieval was accomplished using EDTA (PH 9.0) in super-pressure kettle and endogenous peroxide was blocked using 3% H2O2. After the preparations, slides were incubated in rabbit monoclonal to H3K9me3- Chip Grade (ab8898, diluted 1:1000, Abcam) overnight at 4 °C and then labeled with the EnVision Detection System (Peroxidase/DAB, Rabbit/Mouse) (Dako K5007). Then slides were developed by diaminobenzidine (DAB) and counterstained by hematoxylin. These slides were finally dehydrated and mounted.

IHC-stained sections were digitally imaged using Pannoramic Scanner and CaseViewer software. IHC staining was analyzed by an experienced pathologist and scored for staining intensity (0-no staining, 1-weak, 2-moderate and 3-strong) and percentage of tumor cell staining (0–100). IHC scoring was done by *H*-score which was calculated by multiplying the staining intensity by the percentage of positive cells. Thus, the minimal *H*-score was 0, whereas the maximum *H*-score was 300. To avoid the observation variability, the mean value of duplicate scores was adapted for further analysis.

### Follow-up and outcomes

Patients were followed up by phone calls or out-patient visits every 3 months in the first year, every 6 months in the second and third year after diagnosis and annually thereafter. Outcomes of interest were overall survival (OS) and progression-free survival (PFS). OS was defined as the time from diagnosis to death and PFS was the time from diagnosis to disease progression including recurrence, metastasis and death. The deaths were confirmed by calling the first-degree relatives of the patients and searching the Death Registration Reporting Information System of Guangzhou Center for Disease Control and Prevention. Survival status was censored at the latest follow-up date or Dec 31, 2021.

### Statistical analysis

Wilcoxon signed rank test was used to compare the level of H3K9me3 between tumor tissues and adjacent tissues. Next, the H3K9me3 *H*-score was treated as binary variables. The optimal cut-off value was determined by the minimum *P* value from log-rank chi-square statistics based on PFS using the X-tile 3.6.1 software (Yale University, New Haven, CT, USA) [37]. Chi-square test was used to test the associations of H3K9me3 level with age, menopausal status, histological grade, tumor size, nodal status, clinical stage and expression of ER, PR and HER2. Kaplan–Meier method was used to estimate the 5-year survival. Cox proportional hazard model was used to estimate Hazard ratios (HRs) and their 95% confidence intervals (CIs) for the associations between various prognostic variables and the survival (OS and PFS). Multiplicative scale was used to estimate the interaction between H3K9me3 level and ER status on breast cancer prognosis.

## Supplementary Information

**Additional file 1. Figure S1.** Flowchart of the study cohort. **Figure S2.** X-tile plot of the selected cut-off value for H3K9me3 in tumor tissues. **Table S1.** Univariate association between the demographic and clinicopathological characteristics and the outcomes.

## Data Availability

The datasets used and/or analyzed during the current study are available from the corresponding author on reasonable request.

## Abbreviations

CI: Confidence interval
DAB: Diaminobenzidine
ER: Estrogen receptor
H3K9me3: Histone H3 lysine 9 trimethylation
H3K27me3: Histone H3 lysine 27 trimethylation
HER2: Human epidermal growth factor receptor 2
HR: Hazard ratio
IHC: Immunohistochemistry
OS: Overall survival
PFS: Progression-free survival
PR: Progesterone receptor
TMA: Tissue microarray

## References

[1] Torre LA, Bray F, Siegel RL, Ferlay J, Lortet-Tieulent J, Jemal A (2015). Global cancer statistics, 2012. CA Cancer J Clin.

[2] Bray F, Ferlay J, Soerjomataram I, Siegel RL, Torre LA, Jemal A (2018). Global cancer statistics 2018: GLOBOCAN estimates of incidence and mortality worldwide for 36 cancers in 185 countries. CA Cancer J Clin.

[3] Sung H, Ferlay J, Siegel RL, Laversanne M, Soerjomataram I, Jemal A (2021). Global cancer statistics 2020: GLOBOCAN estimates of incidence and mortality worldwide for 36 cancers in 185 countries. CA Cancer J Clin.

[4] Tang Y, Wang Y, Kiani MF, Wang B (2016). Classification, treatment strategy, and associated drug resistance in breast cancer. Clin Breast Cancer.

[5] Ramadan WS, Talaat IM, Hachim MY, Lischka A, Gemoll T, El-Awady R (2021). The impact of CBP expression in estrogen receptor-positive breast cancer. Clin Epigenet.

[6] Kim HG, Sung JY, Na K, Kim SW (2020). Low H3K9me3 expression is associated with poor prognosis in patients with distal common bile duct cancer. In Vivo.

[7] Song K, Farzaneh M (2021). Signaling pathways governing breast cancer stem cells behavior. Stem Cell Res Ther.

[8] Rogenhofer S, Kahl P, Holzapfel S, Von Ruecker A, Mueller SC, Ellinger J (2012). Decreased levels of histone H3K9me1 indicate poor prognosis in patients with renal cell carcinoma. Anticancer Res.

[9] Ye XD, Qiu BQ, Xiong D, Pei X, Jie N, Xu H (2020). High level of H3K4 tri-methylation modification predicts poor prognosis in esophageal cancer. J CANCER.

[10] Beyer S, Zhu J, Mayr D, Kuhn C, Schulze S, Hofmann S (2017). Histone h3 acetyl k9 and histone h3 tri methyl k4 as prognostic markers for patients with cervical cancer. Int J Mol Sci.

[11] Elsheikh SE, Green AR, Rakha EA, Powe DG, Ahmed RA, Collins HM (2009). Global histone modifications in breast cancer correlate with tumor phenotypes, prognostic factors, and patient outcome. Cancer Res.

[12] Zhao Z, Shilatifard A (2019). Epigenetic modifications of histones in cancer. Genome Biol.

[13] Zhou M, Li Y, Lin S, Chen Y, Qian Y, Zhao Z (2019). H3K9me3, H3K36me3, and H4K20me3 expression correlates with patient outcome in esophageal squamous cell carcinoma as epigenetic markers. Dig Dis Sci.

[14] Benard A, Goossens-Beumer IJ, van Hoesel AQ, de Graaf W, Horati H, Putter H (2014). Histone trimethylation at H3K4, H3K9 and H4K20 correlates with patient survival and tumor recurrence in early-stage colon cancer. BMC Cancer.

[15] Li Y, Guo D, Sun R, Chen P, Qian Q, Fan H (2019). Methylation patterns of lys9 and lys27 on histone h3 correlate with patient outcome in gastric cancer. Dig Dis Sci.

[16] Qian Y, Li Y, Zheng C, Lu T, Sun R, Mao Y (2020). High methylation levels of histone H3 lysine 9 associated with activation of hypoxia-inducible factor 1alpha (HIF-1alpha) predict patients' worse prognosis in human hepatocellular carcinomas. Cancer Genet.

[17] Yokoyama Y, Matsumoto A, Hieda M, Shinchi Y, Ogihara E, Hamada M (2014). Loss of histone H4K20 trimethylation predicts poor prognosis in breast cancer and is associated with invasive activity. Breast Cancer Res.

[18] Zhao QY, Lei PJ, Zhang X, Zheng JY, Wang HY, Zhao J (2016). Global histone modification profiling reveals the epigenomic dynamics during malignant transformation in a four-stage breast cancer model. Clin Epigenet.

[19] Sasidharan NV, El SH, Taha RZ, John A, Ali BR, Elkord E (2018). DNA methylation and repressive H3K9 and H3K27 trimethylation in the promoter regions of PD-1, CTLA-4, TIM-3, LAG-3, TIGIT, and PD-L1 genes in human primary breast cancer. Clin Epigenet.

[20] Kawazu M, Saso K, Tong KI, McQuire T, Goto K, Son DO (2011). Histone demethylase JMJD2B functions as a co-factor of estrogen receptor in breast cancer proliferation and mammary gland development. PLoS ONE.

[21] Gaughan L, Stockley J, Coffey K, O'Neill D, Jones DL, Wade M (2013). KDM4B is a master regulator of the estrogen receptor signalling cascade. Nucleic Acids Res.

[22] Allison KH, Hammond M, Dowsett M, McKernin SE, Carey LA, Fitzgibbons PL (2020). Estrogen and progesterone receptor testing in breast cancer: ASCO/CAP guideline update. J Clin Oncol.

[23] Poon IK, Tsang JY, Li J, Chan SK, Shea KH, Tse GM (2020). The significance of highlighting the oestrogen receptor low category in breast cancer. Br J Cancer.

[24] Luo C, Zhong X, Fan Y, Wu Y, Zheng H, Luo T (2022). Clinical characteristics and survival outcome of patients with estrogen receptor low positive breast cancer. Breast.

[25] Li QL, Lei PJ, Zhao QY, Li L, Wei G, Wu M (2017). Epigenomic analysis in a cell-based model reveals the roles of H3K9me3 in breast cancer transformation. Epigenomics-UK.

[26] Berry WL, Janknecht R (2013). KDM4/JMJD2 histone demethylases: epigenetic regulators in cancer cells. Cancer Res.

[27] Yang J, Jubb AM, Pike L, Buffa FM, Turley H, Baban D (2010). The histone demethylase JMJD2B is regulated by estrogen receptor alpha and hypoxia, and is a key mediator of estrogen induced growth. Cancer Res.

[28] Shi L, Sun L, Li Q, Liang J, Yu W, Yi X (2011). Histone demethylase JMJD2B coordinates H3K4/H3K9 methylation and promotes hormonally responsive breast carcinogenesis. Proc Natl Acad Sci U S A.

[29] Varghese B, Del GN, Cobellis G, Altucci L, Nebbioso A (2021). KDM4 involvement in breast cancer and possible therapeutic approaches. Front Oncol.

[30] Brown LJ, Achinger-Kawecka J, Portman N, Clark S, Stirzaker C, Lim E (2022). Epigenetic therapies and biomarkers in breast cancer. Cancers (Basel)..

[31] Pasculli B, Barbano R, Parrella P (2018). Epigenetics of breast cancer: biology and clinical implication in the era of precision medicine. Semin Cancer Biol.

[32] Baby S, Gurukkala VD, Shankaraiah N (2021). Unravelling KDM4 histone demethylase inhibitors for cancer therapy. Drug Discov Today.

[33] Fu YD, Huang MJ, Guo JW, You YZ, Liu HM, Huang LH (2020). Targeting histone demethylase KDM5B for cancer treatment. Eur J Med Chem.

[34] Sanchez-Munoz A, Perez-Ruiz E, Jurado JM, Ribelles N, Marquez A, Miramon J (2011). Outcome of small invasive breast cancer with no axillary lymph node involvement. Breast J.

[35] He JR, Tang LY, Yu DD, Su FX, Song EW, Lin Y (2011). Epstein-Barr virus and breast cancer: serological study in a high-incidence area of nasopharyngeal carcinoma. Cancer Lett.

[36] Chen QX, Yang YZ, Liang ZZ, Chen JL, Li YL, Huang ZY (2021). Time-varying effects of FOXA1 on breast cancer prognosis. Breast Cancer Res Treat.

[37] Camp RL, Dolled-Filhart M, Rimm DL (2004). X-tile: A new bio-informatics tool for biomarker assessment and outcome-based cut-point optimization. Clin Cancer Res.

