# Supplementary material for: Association of H3K9me3 with breast cancer prognosis by estrogen receptor status
Source: Clin Epigenetics. 2022 Oct 27;14:135. doi: 10.1186/s13148-022-01363-y (PMC9609245; doi:10.1186/s13148-022-01363-y)
Supplement: Supplementary file 1 — Additional file 1. Figure S1. Flowchart of the study cohort. Figure S2. X-tile plot of the selected cut-off value for H3K9me3 in tumor tissues. Table S1. Univariate association between the demographic and clinicopathological characteristics and the outcomes. [file 13148_2022_1363_MOESM1_ESM.doc]

**Supplementary Information**

**Supplementary Figures**


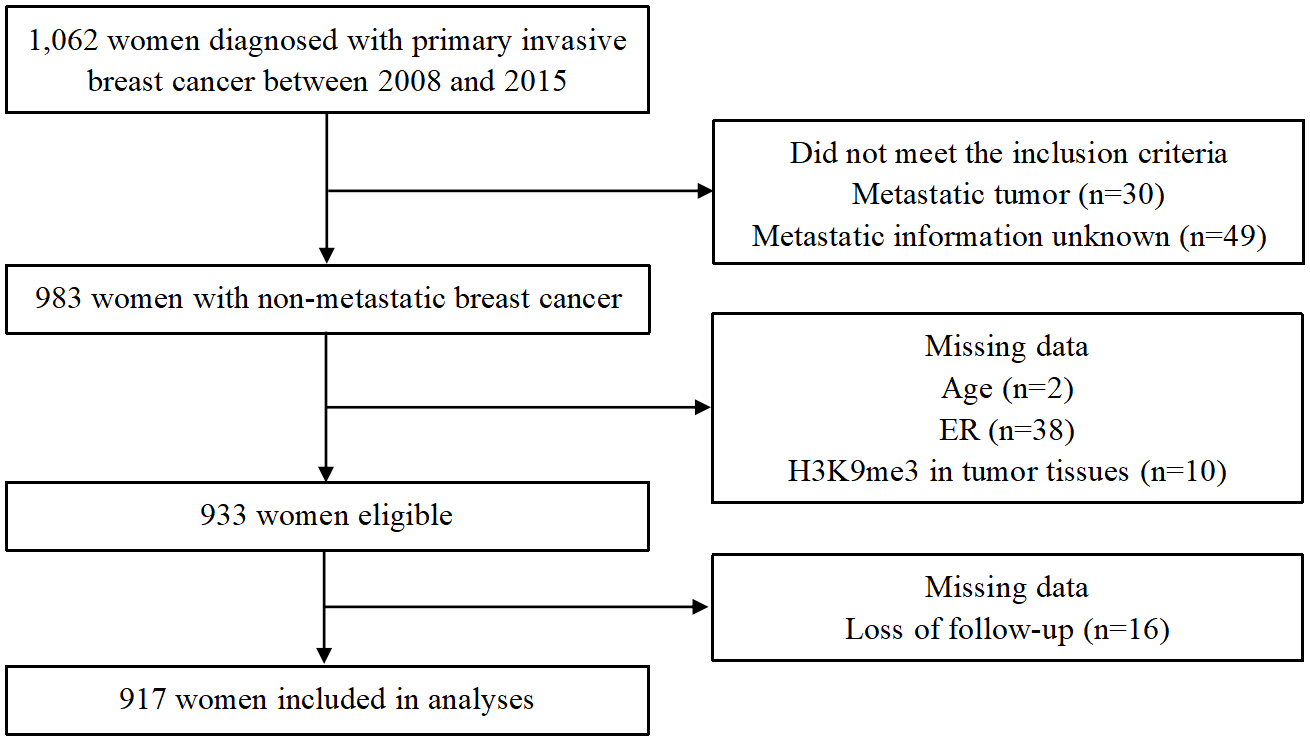


**Figure S1** Flow chart of the study cohort.


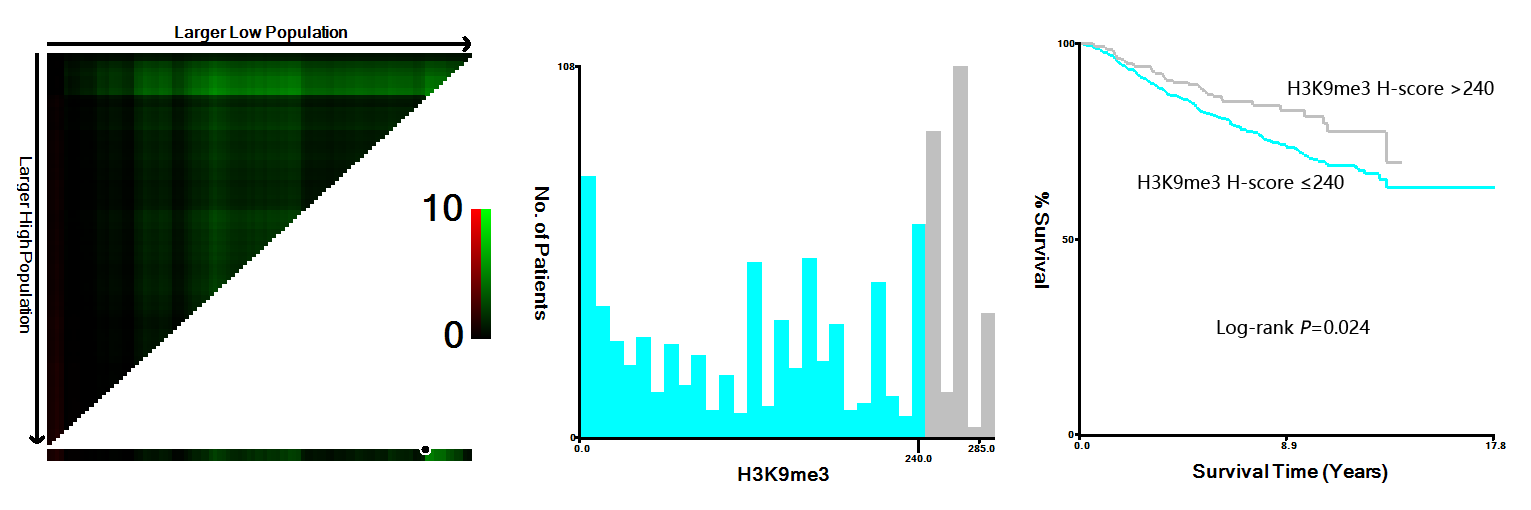


**Figure S2** X-tile plot of the selected cut-off value for H3K9me3 in tumor tissues. In coloration plot, red represents poor prognostic significance of the feature; green represents favorable prognostic significance. Bar graph shows the selected cut-off value. Kaplan-Meier curve shows the relationship between H3K9me3 and progression-free survival.

**Supplementary Tables**

| **Table S1** Univariate association between the demographic and clinicopathological characteristics and the outcomes | | | | | | |
| --- | --- | --- | --- | --- | --- | --- |
| Variables | *N* (%) | OS | |  | PFS | |
|  |  | Event (%) | HR (95%CI) |  | Event (%) | HR (95%CI) |
| Age (years) |  |  |  |  |  |  |
| <40 | 181 (19.7) | 18 (14.2) | 0.69 (0.42,1.15) |  | 045 (22.2) | 1.24 (0.88,1.74) |
| 40-60 | 606 (66.1) | 88 (69.3) | 1.00 (reference) |  | 128 (63.1) | 1.00 (reference) |
| ≥60 | 130 (14.2) | 21 (16.5) | 1.08 (0.67,1.74) |  | 030 (14.8) | 1.07 (0.72,1.60) |
| Menopause |  |  |  |  |  |  |
| Pre- | 506 (57.6) | 60 (48.8) | 1.00 (reference) |  | 103 (53.1) | 1.00 (reference) |
| Post- | 372 (42.4) | 63 (51.2) | 1.40 (0.98,1.99) |  | 091 (46.9) | 1.20 (0.91,1.59) |
| Histological grade |  |  |  |  |  |  |
| I/II | 619 (73.3) | 81 (67.5) | 1.00 (reference) |  | 133 (71.5) | 1.00 (reference) |
| III | 225 (26.7) | 39 (32.5) | 1.40 (0.96,2.06) |  | 053 (28.5) | 1.15 (0.84,1.58) |
| Tumor size (cm) |  |  |  |  |  |  |
| ≤2 | 278 (30.3) | 18 (14.2) | 1.00 (reference) |  | 040 (19.7) | 1.00 (reference) |
| >2 | 639 (69.7) | 109 (85.8) | **2.66 (1.61,4.38)** |  | 163 (80.3) | **1.82 (1.29,2.57)** |
| Nodal status |  |  |  |  |  |  |
| Negative | 412 (44.9) | 33 (26.0) | 1.00 (reference) |  | 058 (28.6) | 1.00 (reference) |
| Positive | 505 (55.1) | 94 (74.0) | **2.60 (1.75,3.87)** |  | 145 (71.4) | **2.35 (1.73,3.18)** |
| Clinical stage |  |  |  |  |  |  |
| I | 165 (18.0) | 06 (04.7) | 1.00 (reference) |  | 017 (08.4) | 1.00 (reference) |
| II | 491 (53.5) | 59 (46.5) | **3.32 (1.43,7.68)** |  | 098 (48.3) | **2.00 (1.20,3.35)** |
| III | 261 (28.5) | 62 (48.8) | 0**7.55 (3.28,17.46)** |  | 088 (43.3) | **3.92 (2.33,6.59)** |
| ER |  |  |  |  |  |  |
| Negative | 247 (26.9) | 43 (33.9) | 1.00 (reference) |  | 063 (31.0) | 1.00 (reference) |
| Positive | 670 (73.1) | 84 (66.1) | **0.64 (0.44,0.92**) |  | 140 (69.0) | **0.73 (0.54,0.98**) |
| PR |  |  |  |  |  |  |
| Negative | 256 (27.9) | 40 (31.5) | 1.00 (reference) |  | 059 (29.1) | 1.00 (reference) |
| Positive | 660 (72.1) | 87 (68.5) | 0.71 (0.49,1.04) |  | 144 (70.9) | 0.82 (0.61,1.11) |
| HER2 |  |  |  |  |  |  |
| Negative | 613 (66.8) | 85 (66.9) | 1.00 (reference) |  | 141 (69.5) | 1.00 (reference) |
| Equivocal | 076 (08.3) | 14 (11.0) | 1.23 (0.70,2.17) |  | 019 (09.4) | 1.05 (0.65,1.70) |
| Positive | 228 (24.9) | 28 (22.0) | 0.94 (0.62,1.45) |  | 043 (21.2) | 0.86 (0.61,1.21) |
| Abbreviations: BMI, body mass index; CI, confidence interval; ER, estrogen receptor; HER2, human epidermal growth factor receptor 2; HR, Hazard ratio; OS, overall survival; PFS, progression-free survival; PR, progesterone receptor.  Significant results (p < 0.05) are shown in bold. | | | | | | |
